# Handwashing among caregivers of young children in a protracted and complex refugee and immigration context: a mixed methods study on the Thai–Myanmar border

**DOI:** 10.3389/fpubh.2023.1099831

**Published:** 2023-07-31

**Authors:** Kasama Pooseesod, Masahiro Umezaki, Athit Phetrak, Suparat Phuanukoonnon

**Affiliations:** ^1^Faculty of Public Health, Thammasat University, Bangkok, Thailand; ^2^Department of Human Ecology, Graduate School of Medical Sciences, University of Tokyo, Tokyo, Japan; ^3^Department of Social and Environmental Medicine, Faculty of Tropical Medicine, Mahidol University, Ratchathewi, Thailand

**Keywords:** water sanitation and hygiene, handwashing, caregivers, refugee, Karen ethnic groups, Thai–Myanmar border, preschool children, qualitative

## Abstract

**Introduction:**

Protracted refugee situations create complex contexts that present significant health risks for young children. Effective hand hygiene practices by caregivers can reduce respiratory infections and diarrhoeal disease, the two largest contributors to mortality among children between 1 month and 5 years of age. This study documented handwashing patterns and access to water, sanitation and hygiene (WASH) infrastructure among caregivers of young children living along the Thai–Myanmar border, one of the world’s most protracted and complex refugee and immigration contexts. It also examined the association between handwashing and socio-demographic variables and captured participants’ explanations for when and how hands are washed. The study broadened the scope of previous research by also including the large number of caregivers living outside formal camps.

**Methods:**

Caregivers of children attending 11 preschools in Tak province, Thailand participated in a mixed-methods cross-sectional study. Quantitative questionnaire data (*n* = 384) were supplemented by a thematic analysis of data from in-depth interviews (*n* = 9).

**Results:**

Fewer than half the caregivers reported routinely washing their hands before preparing meals or after using the latrine/toilet. Fewer than one-in-five routinely used soap in these situations. Interviewees explained that handwashing was only necessary when a substance could be felt or seen, in which case wiping with a cloth or a rinsing with water were sufficient to clean hands. However, their explanations also suggested some potential avenues for culturally appropriate and feasible interventions to improve hand hygiene.

**Conclusion:**

The results confirmed previous research on the multi-dimensional barriers to good hand hygiene in protracted refugee situations and other low-resource settings. Additional investment to overcome shortages in the infrastructure necessary to support good hand hygiene and creative means of drawing on and developing human capital will be necessary to realize the potential hand hygiene holds for reducing ill-health and mortality among young children living in these contexts.

## Introduction

During the past decade, the number of immigrants worldwide almost doubled, with a total of 281 million people living outside their country of birth in 2020 (1). This total includes skilled migrants with entry visas; immigrants with low skill levels (many of whom did not have authority to enter their host country); and by 2022, 108.4 million people who were forcibly displaced by persecution, conflict, violence, human rights violations and events seriously disturbing public order (hereafter referred to as “refugees”) (2).

More than two-thirds of refugees (23.3 million) are currently living in a “protracted situation,” in which more than 25,000 refugees from the same country have lived in exile in the same low- or middle-income host country for at least five consecutive years. In many cases the protracted refugee situation has persisted over multiple generations (e.g., Palestinian refugees in Lebanon, Somali refugees in Kenya, Afghani refugees in Pakistan, refugees from Myanmar in Thailand). In such cases, children born to refugees after their arrival in the host country often also have a precarious legal status. In many cases, the children of irregular immigrants who have spent most or all of their life in the host country experience a similar vulnerability (e.g., *the* “dreamers” generation in the United States).

Many refugees and low-skilled immigrant workers face poorer health outcomes than members of their host communities (3). Refugee and irregular immigrant populations are often exposed to injury and disease in their home country, during transit and after arrival in their host country (4, 5). This is often exacerbated by their vulnerable socio-economic circumstances, limits on their access to medical and social services, and risk factors related to their living and working conditions in their host country. In particular, many refugees and irregular immigrants have living conditions characterised by risk factors for disease including poor sanitation, lack of clean drinking water, low access to preventative health care (e.g., vaccinations), overcrowding, and poor housing (6, 7). Eliminating these underlying causes of disease cannot be accomplished quickly because it requires significant investment and collaboration between multiple stakeholders. However, regular and effective handwashing has proven to be a low-cost preventative measure that can deliver immediate benefits. It can be effective in minimizing the transmission of the most common diseases among refugee populations that have a faecal-oral transmission route (Acute Jaundice Syndrome, Cholera, Giardiasis, Hepatitis E, and Watery Diarrhea) and can also limit transmission of the most common diseases transmitted by air droplets (Influenza Like Illness, Measles, Pertussis, Rubella, and Varicella) by removing any droplets acquired by hand contact with surfaces or an infected person (8).

Previous studies have shown that caregiver hygiene practices are important to support the healthy development of children (9–11). Handwashing with soap can protect children against upper respiratory infections and diarrhoeal diseases (12–15). Globally, these are the most common, and second most common causes of mortality among children between 1 month and 5 years of age (16, 17). Despite this, many caregivers in low- and middle-income countries fail to wash their hands effectively (18–20).

Interventions designed to improve the frequency or adequacy of handwashing often fail (21). The most effective interventions are multifaceted and informed by a theoretical framework (21–23). The factors that influence handwashing are very diverse, and include inadequate awareness or knowledge; forgetfulness; perception that the risk of infection is low; the absence of the physical infrastructure and supplies necessary for handwashing, especially when working outside the home; inconvenience, insufficient time, and a high workload (20, 24, 25). However, different sets of factors appear to influence handwashing behaviour in different contexts. Several guidelines specifically note the need to undertake formative research to identify behavioural determinants among the target population before designing a handwashing intervention (26).

The Thai–Myanmar border region provides a unique opportunity to study water, sanitation and hygiene (WASH) in a complex and protracted refugee and migration context. Thailand is one of forty-four nations that are not party to the 1951 Convention relating to the Status of Refugees and its 1967 Protocol (27). However, since the mid-1980s, refugees have been fleeing across the border into Thailand to escape political repression or armed conflict between ethnic groups and the Myanmar military (28, 29). By 2020, Thailand hosted 85,711 refugees from Myanmar in nine official camps along the border (30). A larger number of refugees from Myanmar have self-settled in the villages of Thai relatives or acquaintances or elsewhere in the border region (31). In Tak province, the majority of these refugees identify as Karen, a diverse ethnic group whose homelands straddle the international border (31). This population of forced migrants has been added to by Karen who enter Thailand as irregular voluntary immigrants seeking a better economic future or access to health care (32). However, because gross human rights violations continue to be perpetuated against Karen living in Myanmar (33), the distinction between forced and voluntary immigrants is often unclear. These populations have been further added to by generations of children born in Thailand to forced or voluntary migrants from Myanmar. Many of these second- and third-generation immigrants have not been granted any legal status in either country (34). Consequently, they are stateless (35). Although about half a million people in Thailand are registered as stateless, some estimates suggest that the real total may be over 2 million (31). The border population also includes Thai citizens whose families migrated from Myanmar prior to the 1980s and Thai citizens who are sheltering refugees and voluntary immigrants from Myanmar in their communities. Only Thai citizens have access to a Thai identity card. Because they lack this card, the other groups face significantly limits on their opportunities to access work, health care and education, and to own land (28). They also experience other disadvantages. Most are poor, relying on subsistence farming in remote mountainous and heavily forested areas (36–38). They belong to an ethnic and linguistic minority group, and are at risk of additional stigma and exploitation due to their vulnerable legal situation (39).

These immigrant populations usually have poor access to health care (3, 28, 39, 40). However, they face significant risk from diseases that have a faecal-oral transmission route (41–43). For example, Mae La refugee camp in Tak province experienced four cholera outbreaks in the 6 years from 2005 to 2010 (41), and in 2020, the prevalence rate for diarrhea in the nine refugee camps in Tak province (61.05 per 1,000) was five times the national prevalence rate (12.56 per 1,000) (44, 45). Despite this, it has proven difficult to increase the frequency or effectiveness of handwashing among caregivers of young children. One example of the challenges is provided by a study of households containing one or more children under 5 years of age living in a long-term camp in Tak province that housed more than 15,000 Karen refugees (46). Behavioural observations and questionnaire data were collected after the International Rescue Committee had implemented a long-term intervention that had two main components: a hygiene education program that included handwashing and was delivered via the camp public address system, house-to-house visits, small group discussions and health education sessions in schools; and provision at no cost of 1 kg of laundry soap and 4 bars of body soap per person to each household every 3 months. As a result of the intervention, water and soap were available at the handwashing stations of almost all households (water 93%; soap 94%) and almost three-quarters of questionnaire participants (73%) correctly reported that it was important to wash hands after using the latrine. Despite this, handwashing occurred in less than half the cases of latrine use or food preparation that were observed, and soap was used in less than 20% of cases. Cultural and personal priorities appeared to play an important role in participants’ behaviour. Most participants reported that laundry and bathing were the most important uses for soap/detergent. It appears that because the allocation of laundry detergent to households was insufficient, households diverted soap intended for handwashing to these tasks. Research in other ethnic minority and rural populations have also demonstrated the influence of personal and cultural priorities and beliefs on caregivers’ hygiene practices (47–50).

Other handwashing interventions along the Thai–Myanmar border have highlighted the importance of context. Far higher levels of handwashing with soap were achieved in a refugee camp in Tak province when the baseline rate of handwashing with soap was already relatively high (66%) and there had been several outbreaks of cholera in the camp (41). A health education program that focused on reducing the prevalence of cholera lifted the prevalence of routine handwashing with soap both 3 months (77%) and 12 months (85%) after the intervention ended.

Most previous research on water, sanitation and hygiene (WASH) in complex and protracted refugee and immigration contexts has been restricted to refugees living in formal camps (51–53). However, fewer than one-in-three refugees live in formal camps (54) and although they are very basic, the WASH infrastructure in camps is often better than can be found in the wider local community. The current research expands the scope of research. It also includes the much larger population of people affected by voluntary and forced migration who live outside camps.

The current research aimed to gain greater insight into the factors that influence handwashing behaviour in complex and protracted refugee and immigration contexts by

1. Documenting patterns of handwashing and access to WASH infrastructure across caregivers living in diverse circumstances;
2. Examining the association between handwashing behaviour and a range of socio-demographic variables; and
3. Capturing explanations for when and how hands are washed reported by caregivers of preschool-age children living along the Thai–Myanmar border.

## Materials and methods

### Study context

**Figure 1:**
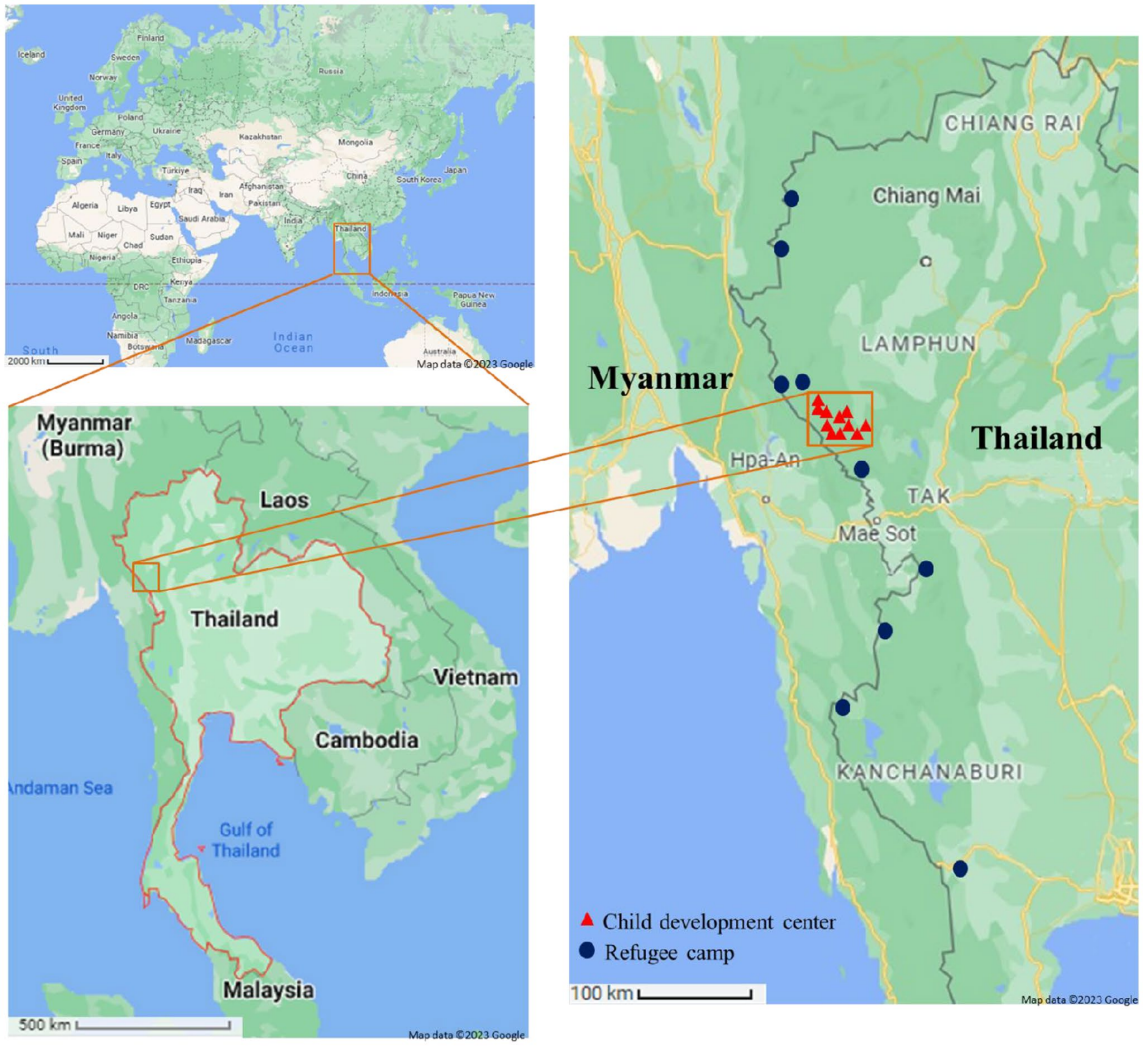
Study location in Tak province, Thailand (55, 56).

From January to June 2018, a cross-sectional study was conducted to assess handwashing practices and associated factors in the Mae Song sub-district of Tha Song Yang District, in Tak province, Thailand (Figure 1). This subdistrict, which lies along the Thai–Myanmar border, contains sixteen villages and is home to approximately 14,000 people.^1^ The Health Register Database of Mae Song health promoting hospital indicates that in 2018 over 88% of the population were members of a Karen ethnic group. According to the Mae Song Sub District Administration Office, approximately a half the population was born outside Thailand, and immigrated from Myanmar. As a result, many Karen have families and friends living on both sides of the international border, and there is regular bidirectional trans-national movement for social visits, in addition to unidirectional movement from Myanmar to Thailand to seek healthcare, work, and education for their children (37). This movement has contributed to the maintenance of homogenous lifestyles and traditions among Karen living on both sides of the border (Permanent Secretary of Mae Song Sub-District Administrative Office, personal communication).

### Sample size and sampling technique

For the quantitative component of the study, participants were recruited from a population of the 417 caregivers of children aged 2–6 years who attending one of eleven child development centers in the Mae Song sub-district. The child development centers run by the Mae Song Sub-District Administration Office (SAO) providing free child care services and early childhood education to all pre-school age children (2–6 years old) living in the SAO administrative areas. In Mae Song SAO, about 96% of pre-school age children attended these child development centers (Permanent Secretary of Mae Song Sub-District Administrative Office, personal communication).

The sample size was determined using the formula of an online statistical calculator [*n* = *z*^2^*p*(1 − *p*)/*e*^2^, where *n* = sample size; *z* = 1.96; *p* = the population proportion; and *e* = 0.05] (32). The population proportion was estimated based on a 2016 survey of handwashing with soap in which 66% of all households living in a long-term refugee camp on the Thailand-Myanmar border were enrolled (41). We calculated the target sample size (*n* = 345) on the basis of 95% confidence intervals (CIs) and a 5% margin of error. Assuming a 15% non-response rate (*n* = 52), achieving the target sample size would require 397 caregivers to be invited to participate. Invitations were issued on the basis of simple random sampling (57). The participation rate (96.7%) was higher than expected: only 13 caregivers declined to participate. The questionnaire was administered to all enrolled caregivers (*n* = 384).

**Figure 2:**
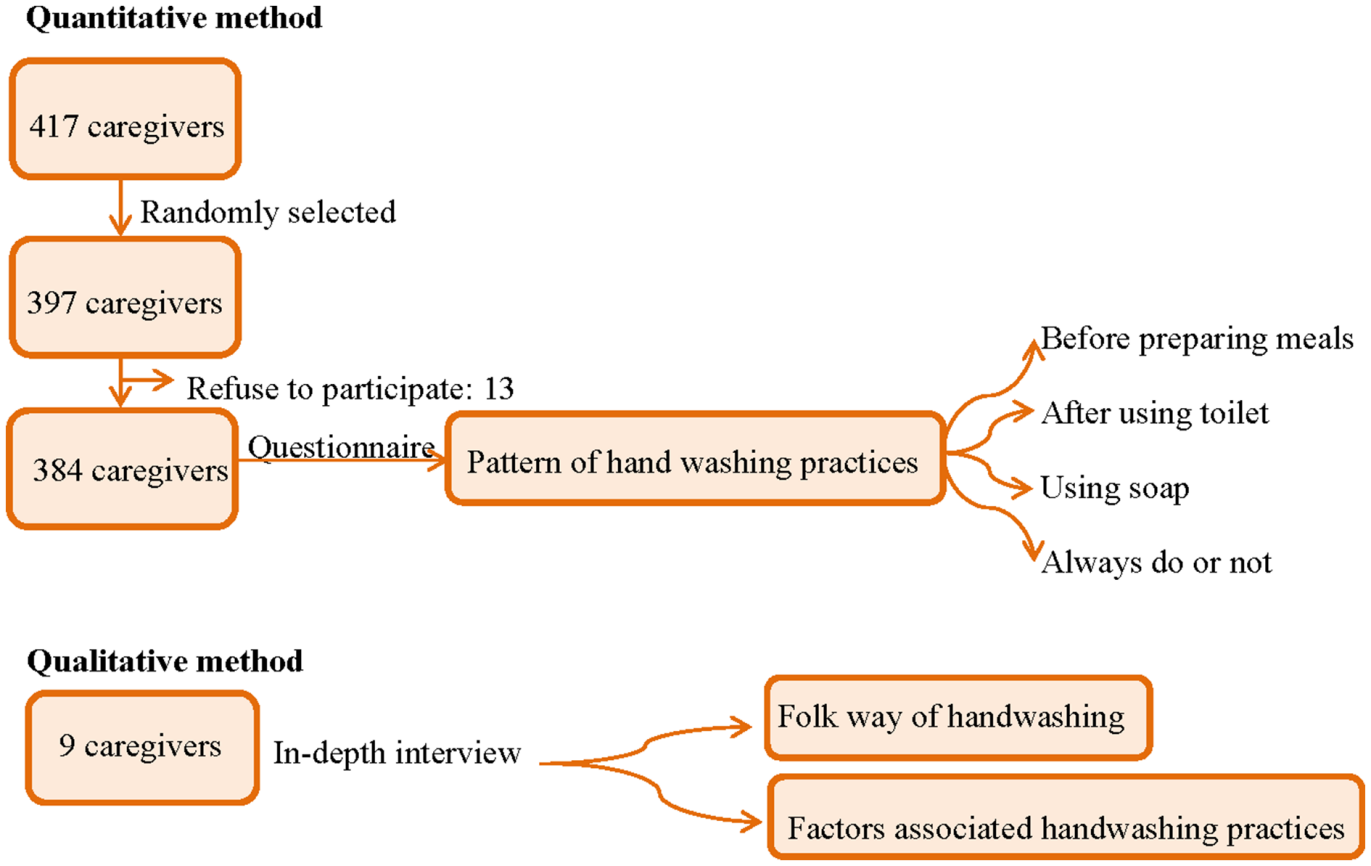
Schematic diagram of the study.

For the qualitative component of the study, we conducted in-depth interviews with a subset of the caregivers of preschool children who had completed the questionnaire and had Thai literacy (*n* = 9). These were recruited via purposive sampling. Community leaders, who served as “gatekeepers,” were consulted to help us identify participants who differed in geographical area, age, gender, and reported handwashing practices (see Figure 2).

### Data collection

Local village health volunteers, who spoke both the Thai and Karen languages, were trained to administer the pencil-and-paper questionnaire. It collected data on three topics: participants’ demographic characteristics (5 questions: age, sex, literacy in Thai language, occupation, and relationship to the child); the socioeconomic characteristics of their household and access to water and sanitation infrastructure (5 questions, materials from which dwelling was constructed; ownership of means of transport; family’s belongings; water source; and type of latrine/toilet); and patterns of handwashing during a typical day (before preparing meals and after using the toilet, with soap/detergent every time or not). The socio-economic characteristics of the household were used to calculate a family wealth index. These scores allowed families to be classified into national quintiles based on wealth (1 = poorest, 5 = wealthiest) (Table 1) (30). To help participants feel comfortable, most questionnaires were completed in their homes. Questionnaires were checked for completeness, quality, and consistency before the data collectors left each house.

**Table 1:** Principal components for the construction of the family wealth index (FWI).

Family wealth index	Housing materials	Transport	Other belongings
1 (Poorest)	Bamboo walls; thatch roof	None	None or chickens
2 (Poor)	Wooden walls; thatch roof	Bicycle	Pigs or goats
3 (Average)	Wooden walls; terracotta roof	Motorcycle	Cattle or horse
4 (Wealthy)	Brick walls; terracotta roof	Tractor	Television set or refrigerator
5 (Wealthiest)	Steel and concrete	Car	Elephant

The goal of the in-depth interview was to gain insights into caregivers’ perspectives on the factors underlying handwashing behaviour in their communities. In doing so, it explored “folk ways,” that is, the socially approved and traditional norms or standards of behaviour in a location (58). The interview guide included prompt questions about factors that influenced handwashing in five specific contexts (before preparing a meal, after using the toilet/cleaning a child who had defecated, before eating a meal/feeding a child, after eating a meal, and after work), and broader factors associated with handwashing practices (e.g., participants’ reasoning about whether soap/detergent was necessary). Interviews took place in locations that were private, convenient and comfortable for the participants. All interviews were conducted by the primary investigator and were recorded with the consent of the participants.

### Analysis and dissemination plan

Quantitative data collected by questionnaires were extracted from the survey database and imported into SPSS software (version 22.0; IBM Corp., Armonk, NY, United States) (59). Categorical variables relating to sociodemographic characteristics and patterns of handwashing were summarized as percentages. A series of univariate analyses examined associations between each sociodemographic factor and whether the participants reported that they always washed their hands before preparing meals and after using the latrine. Variables with *p*-values <0.1 in these analyses were subsequently included in a binary logistic regression. This multivariate analysis estimated adjusted odds ratios (ORs) and corresponding 95% confidence intervals (CIs) for: the caregivers’ relationship to the child: caregiver literacy in the Thai language; caregiver occupation; and urban/rural area of residence. To increase the number of observations in individual categories, caregiver’s occupation and family wealth index were collapsed into dichotomous categories (work inside and outside the household; and low and higher family wealth, respectively). *p*-values <0.05 were considered statistically significant.

Qualitative data collected in interviews were subjected to a deductive thematic analysis (60, 61) in which coding was informed by previous research. In the first step, initial coding of the transcripts was carried out while reading the transcripts line by line (by KP). Related codes were grouped into emerging themes, which were subsequently refined.

Findings were shared with participants, stakeholders, and other interested individuals through community meetings in which residents were able to give feedback to the research team.

## Results

### Quantitative data

#### Socio-demographic characteristics of caregivers

Almost all caregivers were female (*n* = 373, 97.1%) and most were the biological mothers of the preschool-age child who attended the child development/care centres (87.5%). Although almost all caregivers were beyond school age (*M* = 31.9 years, SD = 9.6), fewer than one-third had gained literacy in the Thai language (Table 2). Most caregivers lived in households classified in the lowest two national wealth quintiles even though approximately 79% engaged in some form of out-of-home work, on at least a casual basis. More than two-in five children faced significant health risks because their household had no access to safe drinking water (Table 2). Approximately one-in-ten did not have access to basic sanitation facilities.

**Table 2:** Caregiver and household characteristics for 2 to 6 years-old children living along the Thai–Myanmar border who provided questionnaire data (*n* = 384).

Characteristic	%
*Caregiver*	
Literate in the Thai language	29.2
*Occupation*	
Housework	21.4
Out-of-home work	
Agricultural work (paid or unpaid)	39.1
Casual paid employment	34.1
Government official	3.9
Merchant	1.6
*Household wealth index*	
Lower (national quintiles 1 and 2)	90.1
Higher (national quintiles 3, 4 and 5)	9.9
*Household health risks*	
Source of household water^a^	
Tube well (communal or private)	97.1
Rainwater collection	23.2
Unprotected (e.g., stream, river)	41.4
Unsanitary latrine or open defecation	9.9

a Multiple responses allowed.

#### Handwashing practices

Adequate handwashing practices were reported by only a small minority of caregivers. One aspect of adequacy relates to context. Fewer than one-half of caregivers reported that they always washed their hands before preparing meals or after using the latrine/toilet, and only approximately one-third did both (Table 3). Participants reports showed a high degree of consistency: almost all the caregivers who habitually washed their hands after using the latrine also washed their hands before preparing meals; almost all the caregivers who did not habitually wash their hands after using the latrine also failed to wash their hands before preparing meals. The second aspect of adequacy relates to the use of soap. Fewer than one-in-five caregivers reported always using soap or detergent in either of the key contexts.

**Table 3:** Handwashing practices reported in questionnaires by caregivers of 2 to 6 years-old children living along the Thai–Myanmar border (*n* = 384).

Handwashing practices	%
*Before preparing meals*	
Always washes hands	47.1
Always uses soap/detergent	14.8
*After using latrine/toilet*	
Always washes hands	38.5
Always uses soap/detergent	14.1
*Both before preparing meals and after using latrine/toilet*	
Always washes hands	34.9
Always uses soap/detergent	13.8
Does not always wash hands before preparing meals and after using latrine/toilet	49.3

#### Sociodemographic factors associated with handwashing

Univariate analyses showed that caregiver reports that they always washed their hands after using a latrine/toilet and before preparing meals were associated with four socio-demographic variables: the caregiver’s relationship with the child, literacy in the Thai language, occupation, and area of residence (Table 3). However, the multivariable analysis found that only three of these variables contributed independent variance. Caregivers were at least twice as likely to report that they always washed their hands after using the latrine/toilet and before preparing a meal if they were literate in the Thai language, engaged in home-based work, and did not live in a remote area (Table 4).

**Table 4:** Sociodemographic characteristics associated with handwashing practices among caregivers of 2 to 6 years-old children living along the Thai–Myanmar border (*n* = 384).

Characteristic	*n*	Always wash (%)	Univariate analysis			Multivariable analysis		
			Crude OR	(95%CI)	*p*	Adjusted OR	(95%CI)	*p*
*Caregiver relationship*								
Mother	336	36.9	2.2	(1.1–4.6)	^*^	1.6	(0.8–3.5)	ns
Others	48	20.8	1		—	—		—
*Caregiver age*								
Under 35 years	281	36.3	1.3	(0.8–2.0)	ns			
35 years or more	103	31.1	1		—	—		—
*Caregiver gender*								
Male	11	45.5	1.6	(0.5–5.3)	ns			
Female	373	34.6	1		—	—		—
*Caregiver literacy in Thai language*								
Literate	112	45.5	1.9	(1.2–3.0)	^**^	2.2	(1.4–3.7)	^***^
Illiterate	272	30.5	1		—	—		—
*Caregiver occupation*								
House work	82	46.3	1.9	(1.1–3.0)	^*^	2.0	(1.2–3.3)	^**^
Work outside home	302	31.8	1		—	—		—
*Family wealth*								
Low (FWI: 1–2)	346	34.1	1		—	—		—
Higher (FWI: 3–4)	38	42.1	1.4	(0.7–2.8)	ns			
*Area of residence*								
Remote	326	32.2	1		—	—		—
Not remote	58	50.0	2.1	(1.2–3.7)	^**^	2.3	(1.3–4.2)	^**^

OR, odds ratio; CI, confidence interval; FWI, family wealth index. ^*^p < 0.05; ^**^p < 0.01; ^***^p < 0.001; ns, not significant, *p* > 0.05. ^^^List the variables involved in the multivariate analysis: caregiver relationship, caregiver literacy in Thai language, caregiver occupation, and area of residence.

### Qualitative data

Three themes, each containing two subthemes, were identified in interview data (Table 5).

**Table 5:** Summary of themes in interviews with caregivers of 2 to 6 years-old children living along the Thai–Myanmar border (*n* = 9).

Themes	Subthemes
Knowledge about handwashing	Caregivers’ knowledge
	Children’s knowledge
Beliefs	When hands need to be cleaned
	Effective methods of cleaning hands
Access to necessary resources	WASH infrastructure
	Accessible and affordable soap or detergent

#### Knowledge about handwashing

##### Caregiver knowledge

Knowledge is a necessary but not sufficient condition to support adequate hand hygiene (21, 23). Most interview participants had been told that handwashing could reduce the risk of their children becoming ill, and that using soap improved the effectiveness of handwashing. Some of the caregivers could state the relationship between handwashing with soap and specific health problems among children (e.g., infection by soil-transmitted helminths and diarrhea). Moreover, some caregivers reported being instructed in how to wash their hands correctly by healthcare staff or volunteers when these visited their village, or when they took their children to vaccination centers. For caregivers who could access media (e.g., television, radio, internet), this was also often a source of knowledge about the role of handwashing in disease prevention and control. Caregivers who were literate in the Thai language had greater access to public health information and were more likely to have a Thai identity card that allowed access to health care. Consequently, they were more likely to demonstrate good knowledge of handwashing in interviews:

“I have even learned when and how to wash hands from health staffs who came to the village for providing health education about disease prevention and control such as dengue, diarrhea, and vaccine preventable diseases.” (Woman under 35 years old, Ma Salid Luang Village).

This is consistent with the quantitative questionnaire data, which showed that caregivers who were literate in the Thai language were more likely than those who were not to report that they always washed their hands. Some interview participants also mentioned being encouraged to use good hand hygiene by their local religious leaders, and in some religious traditions, they were required to wash their hands, face and feet before prayers and worship:

“Our pastor from the church supports us to improve personal hygiene. He encourages us to wash hands before prayer, after leaving the latrine, or after eating meals. He also allows us to use his water purifier which located at his home.” (Woman over 35 years of age, Ma Salid Noi Village).

##### Young children’s knowledge

All of the caregivers reported that their children were taught when and how to wash their hands by teachers in child development centers:

“Learning about handwashing practice is more likely to start in the child development centers than at home.” (Man under 35 years old, Mae Kho Village).

However, most caregivers reported that their children were unaware of the benefits of handwashing, and noticed that their children failed to spontaneously wash their hands before consuming snacks in other contexts:

“When we go to work, we let our children stay with their grandparents and play at home. Children can walk to buy snack at the nearest grocery store by themselves and always eat it without handwashing.” (Man under 35 years of age, Ma Salid Noi Village).

The caregivers reported that they reminded their children to wash their hands at home, but usually did so only when their children’s hands were visibly dirty. Consequently, children usually washed their hands before eating meals at home (though not with soap or detergent). Caregivers assisted young children with handwashing, but told children 4 years and older to wash their hands by themselves.

The child development centres also provided children with spoons to limit hand contact with food. Most interview participants reported that they allowed their children to continue to use a spoon to eat meals at home.

##### Beliefs and social norms

Caregivers’ beliefs and the social norms in their community influenced whether and how they implemented handwashing (21, 23).

##### When hands need to be cleaned

Caregivers often washed their hands after finishing daily household chores or returning from out-of-home work. However, many interview participants indicated that they cleaned their hands only in response to a feeling of “dirtiness” (e.g., stickiness or oiliness) or the presence of visually perceptible dirt:

“After getting home from work, I have visible dirt and feel unclean. Then, I wash my hands and feet with only water at the handwashing station (bucket of water) near the stair before entering into the house.” (Man over 35 years old, Mae Song Village).

Similarly, most caregivers reported frequently cleaning their hands with only water both before and after eating meals and feeding children, but did not perceive that their hands needed washing at mealtimes when they could not feel or see anything on the surface of their skin. For example, when the caregivers looked after children during indoor play or while watching television, they thought their hands were not dirty, and therefore, they did not wash their hands before they ate a meal or fed their children.

Handwashing before preparing meals and when feeding children has heightened importance for children’s health in this population since the cultural norm is for food to be eaten with one’s bare hands rather than utensils. This often includes child feeding. One participant stated that Karen people believed that the number of spoons used should be fewer than the number of people eating together, such that they only used a serving spoon when sharing food.

Some caregivers also reported that they washed their hands after using latrine/toilet, and after cleaning a child who had defecated.

“After my child has defecated, I clean his bottom and then wash my hands with only water.” (Woman under 35 years of age, Ma Salid Luang Village).

##### Effective methods for cleaning hands

Cleaning their hands by using cloth to wipe off visible dirt was very common among caregivers, and was perceived to be a quick and convenient method. It was also commonly used to clean visible dirt (typically soil) from children’s hands. When visible dirt prompted handwashing by caregivers, this tended to be done with water alone and for less than 20 s:

“I wash my hands only for cleaning. I use only water to wash my hands. I think it is sufficient to remove dirt and then I feel clean.” (Man under 35 years of age, Mae Nil Khi Village).

“I often wash my hand before eating meals because I use bare hands to eat and I want to clean my hands before. After eating meals, I also wash hands otherwise spicy foods might cause my hands to feel as if they are burning.” (Woman over 35 years of age, Ma Salid Noi Village).

The rationale for the short duration of handwashing was that this was perceived to be all that was necessary to remove dirt and other substances from their skin.

##### Access to necessary resources

Implementation science has demonstrated that interventions to promote behaviour change are most effective when the context makes the right thing to do the easy thing to do. Although access to affordable, reliable and convenient resources does not ensure that these will be used, their absence is a barrier to making handwashing with soap an activity that can be easily integrated into everyday tasks.

##### WASH infrastructure

Caregivers who lived in remote areas were less likely to report that they routinely washed their hands than those who lived in less remote areas. This appears to be attributable to a lack of basic WASH infrastructure in remote villages. In many cases, villagers had access only to a dry pit toilet located between 5 and 20 metres from the main house or needed to resort to digging a hole in the forest for open defecation. Due to a lack of water, wooden sticks or leaves were often used to wipe after defecation, and hands were not washed afterwards. For the same reason, caregivers used wooden sticks to clean their child after defecation and did not wash their hands afterwards:

“My house does not own a private toilet; I have to use the neighbor’s toilet. There is no water in the toilet and I have to use wooden sticks to wipe. I sometimes go to the forest for defecation. I never wash my hand after finish.” (Woman under 35 years old, Mae Nil Village).

For handwashing, washing dishes, and preparing meals, villagers collected rainwater during the rainy season, and during the dry season they used buckets to carry water from the village tube well, where one was available. For tasks requiring large volumes of water (e.g., taking a bath, washing clothes, and watering their vegetable gardens) caregivers in remote villages need to carry water collected from the nearest stream or river. Among the Karen, the heavy physical work involved in supplying the household’s water needs is shared between males and females. More frequent or longer handwashing adds to an already significant burden:

“In rainy season, I have to collect rainwater for washing hands, preparing meals, and washing dishes at the cooking station in front of my house. In dry season, I have to get water from shared tube well of the village for use instead.” (Woman under 35 years old, Mae Nil Khi Village).

In contrast, most caregivers who lived in non-remote areas had access to a sanitary toilet and to their own tube well or pond that provided a year-round source of water. Most used an aqua privy. (An aqua-privy is similar to a single-chamber septic tank, however, the toilet is located directly above the tank. This design reduces the volume of water required for flushing because solids to not need to be moved along a pipe connecting the toilet to the tank.).

However, even when a sanitary toilet was available, some behaviour patterns undermined the hygienic disposal of human waste. Use of toilet paper that can be flushed into an aqua privy is rare due to its cost. Some caregivers who used an aqua privy toilet threw the wooden sticks used for wiping after defecation near the toilet after use. In addition, many caregivers in non-remote locations did not use handwashing practices that are effective in reducing infection: many did not wash their hands; some washed only their left hand (i.e., the hand used for wiping):

“I use aqua privy toilet at home and I use wooden sticks to wipe after finish. I never wash my hands after using toilet because they do not touch faeces.” (Man over 35 years of age, Mae Song Village).

Moreover, soap and detergent use appeared to be rare. When caregivers washed their hands after cleaning a child who had defecated, they used only water.

Caregivers who worked outside the home reported that they rarely washed their hands during work hours, mainly because they did not have access to water outside the home. That is, although caregivers reported that they perceived that their hands were dirtier when working outside the home, and they therefore wanted to wash them, the lack of water in most workplaces precluded this. The extent of this problem varied across workplaces. For example, although most plantations used river or stream water for planting, such water was often not sufficiently clean for handwashing. The problem was most acute for farm workers, most of whom needed to use a pit toilet or dig a hole in the forest for their waste. None of them reported having access to sufficient clean water to allow adequate handwashing:

“When I am employed to harvest on the mountain rice paddies, there is no water and I have to use a little bottled water to wet my hands before eating my packed lunch.” (Man under 35 years of age, Mae Kho Village).

##### Accessible and affordable soap or detergent

Soap and detergent were available at the grocery store in most villages. However, most interview participants reported that they did not routinely wash their hands with soap or detergent because it was expensive, unnecessary and/or inconvenient, even though they did habitually use these products when taking a bath, bathing their children, and washing dishes.

“We are used to washing hands with only water. We rarely use soap/detergent for washing hands because we have to save money.” (Woman over 35 years of age, Thi Mo Ko Tha Village).

“We have to make soap last longer, therefore we use soap only for taking a bath. We use only water without soap to wash our hands.” (Woman under 35 years of age, Mae Nil Village).

“I do not like to use soap/detergent for washing hands because it was not convenient for me. It takes time to scrub hands with soap/detergent and then rinse hands with water.” (Woman under 35 years of age, Mae Nil Village).

In addition, participants who worked outside the home indicated that they did not have access to soap or detergent during their work:

“When I go outside home, I never carried soap/detergent. I use only water to wash my hands.” (Man under 35 years of age, Ma Salid Noi Village).

Thus, most caretakers in this study used only water when they washed their hands. However, this was not the case in traditional Karen culture. In the past, when soap was not available, it was common for ash or specific local plants to be used as cleaning agents. There is little scientific research on the efficacy of the most commonly used plants: wild snake gourd (*Trichosanthes cucumerina* L.) and sesame leaves. Today, this tradition continues among some elders, but is rare among younger generations:

“My mother has ever told me that she used to use stone for body scrub when taking a bath at the river because soap was not available in the past. She used snake gourd or sesame leaves for washing hand, washing hair, and washing clothes, and she used ashes for washing dishes.” (Woman over 35 years of age, Ma Salid Noi Village).

“My parents still never use soap for taking a bath and washing hand. On the contrary, I not only use soap but also use facial foam and shampoo.” (Woman under 35 years of age, Ma Salid Luang Village).

Although Karen communities in Myanmar generally have poor health care (62), interview participants who cross the border indicated that handwashing facilities (i.e., soap, detergent and water) were available here:

“When I go to Myanmar for visiting my relatives, I can wash my hands like I do at home. My relative’s house had water and soap/detergent for use as well as for hand washing.” (Woman over 35 years of age, Thi Mo Ko Tha Village).

## Discussion

This study addressed three main aims. First, it collected quantitative data on patterns of handwashing and access to WASH infrastructure among caregivers of preschool children living in the complex and protracted refugee and immigration context along the Thai–Myanmar border. Unlike most previous research, this study also encompassed caregivers dispersed outside formal refugee camps. Fewer than one-in two caregivers routinely washed their hands after using the toilet/latrine or before preparing meals and only about one-in-seven caregivers washed their hands using soap or detergent. This suggests that young children living across a wide area along the border are at elevated risk of the faecal-oral transmission of disease that has led to repeated episodes of cholera (41) and diarrhea (44) in some refugee camps in the area. However, it also found that over 40% of young children lived in households with a water source that was not protected from contamination by human or animal faeces. This suggests that although improved hand washing may decrease the risk of disease for some children, this alone will not be sufficient to protect many children from diseases with a faecal-oral transmission route. The study’s second aim was to examine the association between handwashing behaviour and a range of socio-demographic variables. These analyses had the potential to identify both groups within the population who could serve as models of appropriate handwashing practices and groups whose children may be at highest risk of illness. Only three socio-demographic variables showed independent positive associations with whether caregivers reported that they routinely washed their hands after using the latrine/toilet and before preparing meals: literacy in the Thai language, work in the home, and residence in an area that was not remote. The study’s third aim was to capture caregivers’ explanations for when and how hands should be washed in order to investigate folk methods of handwashing. The results indicated that many participants had been instructed in appropriate handwashing methods, and that they had been told that it was important to wash their hands after using the latrine/toilet. However, few participants implemented this knowledge because it was inconsistent with their belief that it was only necessary to wash their hands when they could feel or see material on their hands, and that briefly rinsing their hands with water alone was sufficient to remove this material. Similar belief patterns have been reported by caregivers in diverse low- and middle-income countries and have proven to be a significant barrier to the uptake of routine handwashing with soap and water (18, 20, 24, 63).

### Potential strategies for intervention

Previous research has shown that interventions to improve caregiver handwashing are effective when they do not attempt to convince caregivers of the existence of “invisible dirt” in the form of germs, and instead focus on integrating the desired behaviours into caregivers’ existing beliefs, habits and priorities (22). The qualitative data produced by this research suggested several directions that could be explored in custom-designing an intervention of this type for the population living along the Thai–Myanmar border. First, many of the participants in the current study routinely cleaned visible dirt from their hands with a cloth. It may be possible to adapt this existing habit into one that is effective in achieving hand hygiene by using a micro-fibre towel with an anti-microbial treatment. When dipped in water, the “Supertowel” effectively kills pathogens on hands. Eritrean refugees living in a camp in Ethiopia reported that it was convenient, easy to use, saved them water and money and was more desirable than alternative hand cleaning products (64).

Second, it may be possible to develop interventions based on traditional Karen hygiene practices. Participants reported that older adult Karen used ash or specific local plants as hand cleaning agents and/or when bathing. There appears to be no research on the disinfecting properties of the most commonly used local plants. Ash is a traditional cleaning agent that continues to be widely used in communities in low- and middle-income countries in which soap has low affordability, accessibility, or acceptability. Handwashing with soap is promoted because it is an effective means to an end: removing contamination that can cause illness from hands. However, many studies have shown that when wood ash is stored using methods that avoid its contamination and is rinsed from hands with sufficient water, it can be as effective as soap in removing pathogens and in reducing the incidence of moderate to severe diarrhea in young children (65–68). Moreover, those studies that have found soap to be superior to ash, nevertheless report that handwashing with ash reduces pathogens far more effectively than water alone (69). These findings raise the possibility that elders in the community could contribute to the reduction in illnesses among young children by acting as agents of change to reinstate, and affirm the value of, a traditional cultural practice that uses a cleaning agent that is widely available and requires no additional expenditure. This might be an end goal or a preliminary step that established the habit (70) of washing hands with a cleaning agent (63, 71) before attempting to change this agent to soap.

In addition, caregivers are not the only agents of change who can be effectively targeted in hand hygiene programs to support children’s health. Because young children frequently place their hands near or in their mouths, their hand hygiene is also important for the reduction of diarrhea and other illnesses (72) Several interventions have sought to improve young children’s hand hygiene by increasing the frequency or effectiveness with which they wash their own hands (73). Many of these interventions have been designed for children living in high-income countries. However, several studies have been conducted in low-resource settings. For example, an intervention that provided infectious disease risk communication and used puppetry and model-making improved handwashing with soap by children in an informal settlement in Nairobi, Kenya (74). In addition, two studies have been effective in increasing handwashing among the children of forced migrants. In a camp for internally displaced people in Iraq, children provided with transparent soap in which a toy was imbedded were four times more likely to wash their hands with soap after key events than children in the control group, who received plain soap (75). However, providing children with either type of soap was effective in increasing handwashing with soap in a camp for internally displaced people in Somalia, where the base rate of handwashing was lower and the intervention was delivered to older children (76). In the current study, respondents indicated that children routinely washed their hands with soap at their child development/care centers. However, the social norm this created was not transferred to contexts outside the centers. Previous interventions (22) that successfully created and maintained community-wide “hygienic social norms” may provide insights into strategies that may achieve this in communities along the Thai–Myanmar border.

### Access to resources required for hand hygiene

Both theoretical frameworks for behaviour change in handwashing (21, 23) and empirical evidence (12, 13, 22, 73, 77) indicate that multifaceted interventions that enhance enablers and overcome barriers are needed to create sustained change in hand hygiene behaviors. However, a commitment to routine washing hands with soap cannot be enacted without access to soap and clean water. The caregivers in the current study living in remote areas were less likely to routinely wash their hands than those living in non-remote areas. This can be at least partly attributed to a lack of access to their own water sources. This is particularly significant, since families in remote areas also had the lowest access to sanitary toilets and are less likely to have easy access to healthcare facilities. As in previous studies (24, 25, 78), many of participants cited a lack of physical infrastructure and supplies as important barriers to handwashing. This is a widespread challenge for efforts to reduce childhood illnesses with a faecal-oral or airborne droplet route of transmission. For example, according to the WHO/UNICEF Joint Monitoring Programme for Water Supply, Sanitation and Hygiene report (79), two in every five people in South-East Asia do not have soap and water on their premises for handwashing. Moreover, improvements in handwashing facilities and behavior will not realize their full potential in reducing childhood illness as long as a large percentage of their households continue to rely on unprotected water sources.

Previous research in refugee camps along the Thai–Myanmar border suggest that laundry and bathing are priority uses for soap (46). It is unlikely to be helpful to increase handwashing with soap by diverting its use from these contexts since removing pathogens from clothing and skin are also important strategies for supporting children’s health. Removal of pathogens from clothing is particularly important in contexts in which hands are dried on clothing (80, 81), since this can re-contaminate clean hands (67), or where poor sanitation facilities can lead adults’ and children’s clothing to be soiled by urine, faeces, or soil containing helminths.

### Limitations

The findings of this study should be interpreted in the context of its limitations. First, both quantitative and qualitative data on handwashing patterns were self-reported and may have been influenced by a social desirability bias (82). Ideally, they would be supplemented by behavioral observations conducted in a way that minimizes observer effects. Second, due to the sensitivity of issues concerning legal status and identity, the study did not gather information about the caregivers’ length of residence in Thailand, or about whether they were Thai citizens, refugees, voluntary immigrants, or stateless people. This precluded analyses that may have identified important differences in young children’s exposure to health risks among these groups.

## Conclusion

Sustainable Development Goal 3.2 targets the elimination of preventable mortality among children under 5 years of age. Protracted refugee situations present a challenge to the achievement of this goal. At the end of 2022, there were 57 protracted refugee situations in 37 different host countries (2). Most of these were low- or middle-income countries with limited health infrastructure. The two conditions that make the greatest contribution to child mortality between 1 month and 5 years of age are respiratory infections and diarrheal disease (16, 17). Routine effective handwashing by caregivers can make an important contribution to reducing both of these conditions. However, this research has demonstrated some of the multi-dimensional challenges to establishing and maintaining this behaviour in a complex and protracted refugee and immigration context. Notwithstanding this, the study has also shown how formative research that identifies the determinants of hand hygiene behaviors among a target population can suggest innovative custom-designed strategies for intervention. The potential benefit of such behavioral interventions can only be realised if caregivers have access to culturally appropriate, affordable, and accessible sources of clean water and cleaning agents, and safe means of disposing of human waste. All of these are currently in short supply in many complex and protracted refugee and immigration contexts, including the one studied in this research.

## Data availability statement

The original contributions presented in the study are included in the article/supplementary material, further inquiries can be directed to the corresponding author.

## Ethics statement

The studies involving human participants were reviewed and approved by the Ethics Committee, Faculty of Tropical Medicine, Mahidol University, Thailand. The patients/participants provided their written informed consent to participate in this study.

## Author contributions

SP, AP, and MU conceived and designed the study. KP, AP, and SP conducted household survey, data management, analysis, and interpretation of data. KP and SP drafted the manuscript. All authors contributed to the article and approved the submitted version.

## Conflict of interest

The authors declare that the research was conducted in the absence of any commercial or financial relationships that could be construed as a potential conflict of interest.

## Publisher’s note

All claims expressed in this article are solely those of the authors and do not necessarily represent those of their affiliated organizations, or those of the publisher, the editors and the reviewers. Any product that may be evaluated in this article, or claim that may be made by its manufacturer, is not guaranteed or endorsed by the publisher.

## Footnotes

^1^https://en.wikipedia.org/wiki/Tha_Song_Yang_district
