# Corrigendum: Handwashing among caregivers of young children in a protracted and complex refugee and immigration context: a mixed methods study on the Thai–Myanmar border

**DOI:** 10.3389/fpubh.2023.1279605

**Published:** 2023-08-29

**Authors:** Kasama Pooseesod, Masahiro Umezaki, Athit Phetrak, Suparat Phuanukoonnon

**Affiliations:** ^1^Faculty of Public Health, Thammasat University, Bangkok, Thailand; ^2^Department of Human Ecology, Graduate School of Medical Sciences, University of Tokyo, Tokyo, Japan; ^3^Department of Social and Environmental Medicine, Faculty of Tropical Medicine, Mahidol University, Ratchathewi, Thailand

**Keywords:** water sanitation and hygiene, handwashing, caregivers, refugee, Karen ethnic groups, Thai–Myanmar border, preschool children, qualitative

In the published article, there was an error in affiliation(s) [Athit Phetrak^1^]. Instead of “Athit Phetrak^1^”, it should be “Athit Phetrak^3^”.

The authors apologize for this error and state that this does not change the scientific conclusions of the article in any way. The original article has been updated.

